# Laser-Generated Proton Beams for High-Precision Ultra-Fast Crystal Synthesis

**DOI:** 10.1038/s41598-017-12782-w

**Published:** 2017-10-02

**Authors:** M. Barberio, M. Scisciò, S. Vallières, S. Veltri, A. Morabito, P. Antici

**Affiliations:** 1 INRS-EMT, 1650 Boul. Lionel Boulet, Varennes, Canada; 2 INFN and University of Rome, Via Scarpa 14, 00161 Roma, Italy; 3 ELI-ALPS, Secondary Sources Division, Tsiza Lajos krt, 85-87, Szeged, Hungary

## Abstract

We present a method for the synthesis of micro-crystals and micro-structured surfaces using laser-accelerated protons. In this method, a solid surface material having a low melting temperature is irradiated with very-short laser-generated protons, provoking in the ablation process thermodynamic conditions that are between the boiling and the critical point. The intense and very quick proton energy deposition (in the ns range) induces an explosive boiling and produces microcrystals that nucleate in a plasma plume composed by ions and atoms detached from the laser-irradiated surface. The synthesized particles in the plasma plume are then deposited onto a cold neighboring, non-irradiated, solid secondary surface. We experimentally verify the synthesizing methods by depositing low-melting-material microcrystals - such as gold - onto nearby silver surfaces and modeling the proton/matter interaction via a Monte Carlo code, confirming that we are in the above described thermodynamic conditions. Morphological and crystallinity measurements indicate the formation of gold octahedral crystals with dimensions around 1.2 μm, uniformly distributed onto a silver surface with dimensions in the tens of mm^2^. This laser-accelerated particle based synthesis method paves the way for the development of new material synthesis using ultrashort laser-accelerated particle beams.

## Introduction

The field of laser-accelerated proton beams, produced during the interaction of a solid target with a high-intensity (I > 1 × 10^18^ W/cm^2^), short pulse (<1 ps) laser, is a domain of increasing attractiveness in particular for the unique properties that these beams feature^1–4^. Today, typical proton beams that can be routinely obtained on high-power lasers exhibit about 10^13^ particles per shot, are ps duration at the source, have an energy in the tens of MeV^5^ and very good laminarity^6^. While strong effort is put to materialize different applications such as in fusion^7^, radiography^8^, astrophysics^9,10^, neutron production^11,12^, medicine^13^, cultural heritage^14^, or novel particle injector^15,16^, material science applications are still in a very embryonic state while a strong claim is made to explore this new field of endeavor^17^. Some of the unique features of laser-driven protons, in particular short-duration and high flux, have the potential of improving many conventional applications where these parameters are important and represent a bottleneck. In material science, one potential field facing strong challenges is the synthesis and growth of nano/micro-crystals and structured surfaces: Empirical procedures have been developed for the preparation of a wide range of semiconductor, ceramic, and metallic nano- and microcrystals, where the methods as well as growth parameters are often very specific to a single research group. Presently, achieving fine control over the monodispersity, structure, composition and defects of micro/nanoparticles is still a major challenge in micro- and nanotechnology and is tackled by many research groups since considered strategically important for manifold applications^18–20^. The ability to generate nano- and microstructures with a high-precision technique allows improving applications in several fields. In medicine, particles with dimensions ranging from sub-ten nm up to a few tens of nm can be used for enhancing imaging techniques such as Raman spectroscopy and magnetic resonance^21^. However, the high-precision manufacturing of these particles, solvent-free, is still challenging, and new techniques for developing them are strongly in demand^22^. Particularly in the biomedical field, higher-quality results can easily justify more expensive techniques when it comes to overcome a critical problem that cannot be solved differently (an example is the proton therapy, very expensive tumor treatment, yet unique for curing particular types of tumors^23^).

However, the quest for high-precision crystals is not only made in biomedical applications. Micro- and nanoparticles influence the hydrophobic and optical properties of biological^24,25^, and architectonical devices^26^, additionally, it has been demonstrated that nanostructured materials used in photovoltaic applications have the ability to alter the electrical cells’ properties and increase the overall efficiency of photovoltaic devices^27,28^. The main problem in the definition of a standard growth protocol for these nano- or microcrystals is identifying the parameters to generate the conditions of temperature and pressure that are required to produce well-defined structures in a very short temporal range (ps-ns). These short timescales are necessary for the nucleation of particles with dimensions of up to a few nm, where conventional techniques currently lack in precise manufacturing techniques. A way to achieve this is the irradiation of matter by an energetic proton beam with short duration. The irradiation of a bulk target by high-energetic short-pulse protons, such as generated by interaction of a high-power laser with a solid target, can generate the temperature and pressure conditions required to grow crystalline structures^9,29^.

In this paper, we introduce a physical method for the synthesis of micro-crystals and microstructured surfaces based on the ablation of material using an ultra-short ultra-intense laser-generated proton beam. In our synthesis process a solid surface material having a low melting temperature is ablated by irradiation of a high-energy short-pulse laser-accelerated proton beam. Compared to conventional ablation technologies, in our method the sample is intensively irradiated for only a few ns, reaching thermodynamic conditions that are between the boiling and the critical point. The high-energy proton beams are generated by the acceleration mechanism commonly known as Target Normal Sheath Acceleration mechanism (TNSA)^30^ occurring when a high-intensity (I > 10^18^ W/cm^2^), short-pulse (duration <1 ps) laser hits a target with micrometric thickness under vacuum conditions. The laser-generated protons irradiate a second sample, generating in its bulk temperature and pressure conditions that are unreachable in conventional nanomaterial laboratories using industrially produced ion beams^9,29^. These conditions favor the nucleation of micro-crystals with a good control in crystallinity and dimensions^31^. The interaction between the laser-generated proton beam and the low-boiling material causes a mechanism that is similar to the conventional Laser Ablation, detaching the atoms and ions from the target surface and producing the formation of micro-particles with very high mean energy within a plasma plume. These particles are then deposited on nearby cold solid surfaces producing nano- and microstructuration.

**Figure 1:**
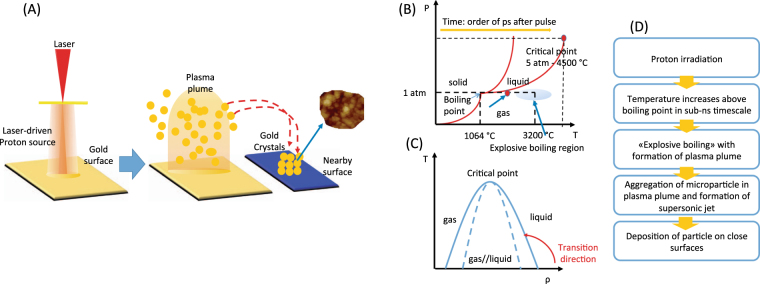
(**A**) Sketch of the micro-structure synthesis process produced by Laser-Driven Proton Ablation, (**B**–**D**) explained in terms of a classical thermodynamic model. ﻿P indicates the pressure, T the temperature, ρ the density.

The Laser-Driven Proton Ablation (LDPA) occurs in three steps (described in Fig. 1A,B): (i) the synthesis process starts with the interaction between the high-energy proton beam and the bulk target, interaction occurring in a ps-ns timeline. (ii) The target bulk heats up, in a few hundreds of ps, to temperatures ranging between the boiling and the critical point (see pressure-temperature phase diagram in Fig. 1B). The penetration depth of the impinging protons (in our case protons with a maximum energy ranging up to 20–30 MeV) is in the order of tens of microns and allows for an in-depth heating of the irradiated sample up to this distance. (iii) A plasma plume containing the ablated materials expands into the surrounding vacuum and cools down; In the plasma plume generated by the laser-accelerated protons, the ablated materials (i.e., atoms, ions, nanocluster, etc…) nucleate, forming micro-structures which are deposited onto the cold surrounding surfaces reached by the plasma plume.

In the first phase, the detachment of the atoms and ions from the original target surface starts with the interaction between the laser-accelerated protons and the material’s bulk and lasts over a timespan up to the tens of ns. This is longer than the proton beam irradiation (in our case, the heating process lasts a few ns, considering the proton energies that mostly contribute to the heating of the sample’s surface) since it takes time to transfer the thermal energy deposited by the protons. The short plume duration generated by the laser-accelerated protons limits the nucleation time to the range of ns, ensuring the stop of nucleation in the phase where atoms arrange in crystals or nanoparticles without aggregation of amorphous structures. The nucleation of a nanoparticle in a hot plasma starts in the first ps of the plume formation and continues until the plume cooling phase has finished (i.e. the surface temperature has dropped below the boiling point) and the deposition on the secondary surface has occurred. As reported in the section devoted to the numerical results of this study, in our specific case the plume has  a duration in the tens of ns and the micrometric dimensions of the obtained gold crystals are typical of a cooling phase of about 100 ns. Despite this longer cooling phase, the elapsed time is still sufficiently short to prevent reaching the condensation phase, which takes place over a time span longer than hundreds of ns; additionally the quick cooling avoids the formation of larger and amorphous particles in the plume. Given the stringent temperature conditions, the proton-induced heating occurs only in the region that is almost corresponding to the spot irradiated by the proton beam, i.e. an area in the range of mm^2^. One can see that the proton heating duration is critical for the crystal generation. As such, any variation of the interaction conditions between the laser-accelerated proton beam and the irradiated material sample (e.g. changing the number of protons per unit of irradiated surface, varying the distance between the particle source and the sample or tuning the length of the laser pulse for achieving different irradiation times of the sample), that results in a modification of the heating time, might potentially change the features of the generated crystals. The influence of these parameters therefore allows controlling the features of the obtained particle structure (as will be discussed in a future publication) and, with appropriate tuning, shall allow for generating nanostructures reaching dimensions in the nanometer scale. The generated nano- or microparticles can be easily detached from the surface, e.g. by performing a sonication of a few minutes in a polar solvent bath, for mixing them into a colloidal solution.

We can model the ablation mechanism using in a first approximation a classical thermodynamic model. In our synthesis method, only three thermal processes may lead to the material removal from a proton-irradiated target: vaporization, normal boiling and explosive boiling. In the case of high-energy, short-pulse proton beams, the explosive boiling can be considered as being the main mechanism for the synthesis process. It occurs when solid matter is rapidly (in a timeframe in the order of ps-ns) superheated to temperatures higher than the boiling point. In these conditions a spinal decomposition takes place in the vapor and liquid phase, accompanied by a homogenous nucleation. The thermodynamic evolution of the irradiated target, going from solid to explosive boiling, can be described using the temperature-density phase diagram shown in the Fig. 1C: the fast heating and concomitant density drop leads the system to the region of spinodal decomposition where the explosive boil occurs (the evolution of the system is indicated by a red arrow in Fig. 1C). The plasma plume is confined to the region where the temperature is higher than the boiling point. Different physical phenomena occur on the material surface from where the plume is generated: the surface of the irradiated area melts during the heating timeframe and the solid lattice of the material is completely destroyed. During the cooling phase the atoms on the surface rearrange their position, forming uncontrolled structures (amorphous aggregation, micro- and nanoparticles). These aggregation characteristics can be very different from case to case and strongly depend on the temperature and on the duration of the cooling phase. In our case, experimental results indicate a complete melting of the gold surface, which after irradiation appears completely destroyed and covered by amorphous gold droplets (see later).

In order to confirm the above described synthesis process we performed experiments on the TITAN laser of the Jupiter Laser facility (Lawrence Livermore National Laboratory - LLNL) (see experimental setup details in the Supplementary Materials - methods section). This laser produces pulses of about 220 J in 700 fs and operates at a wavelength of 1.053 µm^32^. The proton spectra obtained by this laser are a reproducible and measurable benchmark of laser-driven proton production on a high-power laser facility of last generation^33^. A typical laser-generated proton spectrum such as that obtained and used during the experiment is shown in the method section. The laser-generated protons were impinging a commercially available solid gold target with dimensions of 5 × 15 mm and of thickness 100 µm located at a distance of 2.5 cm from the proton source (see methods section). Two silver targets of dimensions 2.5 × 10 mm were placed at both sides of the gold sample in order to catch all the nanoparticles generated by the gold target in the plasma plume. The silver targets were touching the gold target at one end while at the other end they were distant of 4 mm.

**Figure 2:**
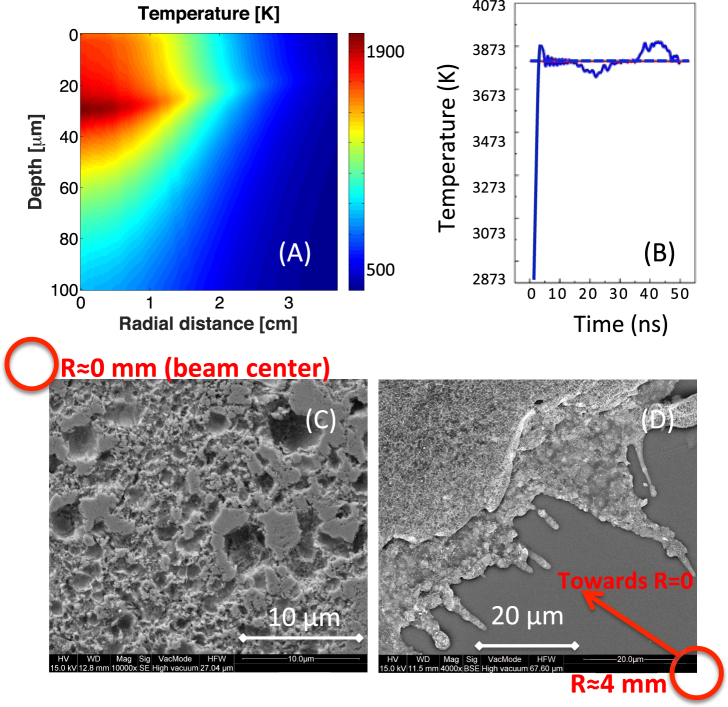
(**A**) Computed temperature map of the gold sample, about 900 ps after irradiation, The target is located at 2.5 cm in order to adapt the proton flux to the requested temperature conditions. The 0 level indicates the target surface in front of the proton beam; (**B**) Temperature evolution in time, monitored on the target surface. The temperature has been computed in the warmer region at a penetration depth of 40 μm and at a radial position of nearly beam center. The maximum temperature shown in Fig. (**A**) does not correspond to the maximum temperature as found in Fig. (**B**) since in Fig. (**A**) the sample temperature is still in the onset phase. After the onset, the temperature stays above the boiling point for tens of ns, ensuring the duration of the plume. The blue dashed line is a guide for the eye showing the mean value. (**C**) SEM image of the gold target that has been irradiated by the laser-generated protons, image taken near the beam center that is indicated with a red circle. (**D**) SEM image of the same target as in (**C**) but at a beam distance of 4 mm (indicated with a red circle), the beam center direction is indicated with a red arrow.

We performed preliminary simulations with the above-mentioned laser and proton parameters in order to verify and optimize the synthesis conditions. The proton-target interaction was modeled with a Monte Carlo code, in which we inserted as heating source the laser-generated proton beam obtained on the TITAN laser. The use of this kind of code is a standard in the field of laser-plasma interaction for measuring proton-induced heating and pressure effects. Figure 2A shows the computed temperature map obtained for a 100 µm thick monocrystalline gold foil irradiated by the laser-generated proton beam obtained on the TITAN laser. The temperature map was collected earlier than 1 ns after irradiation and for a target located at an optimized distance of 2.5 cm from the source. The computed temperature (Fig. 2A) indicates that the bulk target heats up to temperatures higher than the boiling point in almost the entire region covered by the proton beam (area with a radius of about 4 mm and depth of 80 μm). From simulations we also see that the plasma pressure stays constantly below 500 kPa in the entire target bulk, excluding the possibility to reach the thermodynamics critical conditions for gold, which are 4800 K at 500 kPa (5 atm). The density is almost constant all over the target and is comprised between 18.5 and 19.1 g/cm^3^ in the entire region covered by proton beam. These values are lower than the gold solid state (19.33 g/cm^3^).

The numerical results presented in Fig. 2A show that in the current setup the gold target irradiated by the laser-generated proton beam reaches the thermodynamic conditions for explosive boiling. This is confirmed by SEM images shown in Fig. 2C and Fig. 2D, that display the melted surface of the gold target at the beam center and up to a radial distance of 4 mm. The above-mentioned findings strengthen the predicted evolution of our system (consisting of the irradiated target) as foreseen by our model (red line in Fig. 1C).

We also observe, from temperature/time profiles in Fig. 2B that the temperature (taken in the warmer region at a depth of 30 μm) stays constant above the boiling point for at least 50 ns after irradiation, timespan corresponding approximately to the duration of the gold plume. The ablation time, lasting tens of ns, therefore allows the formation of aggregates in the plume, with dimensions in the few to tens of microns, accompanied by a massive erosion of gold from the target and the deposition of a thick layer of gold nanoparticles on the surface of the nearby silver target.

**Figure 3:**
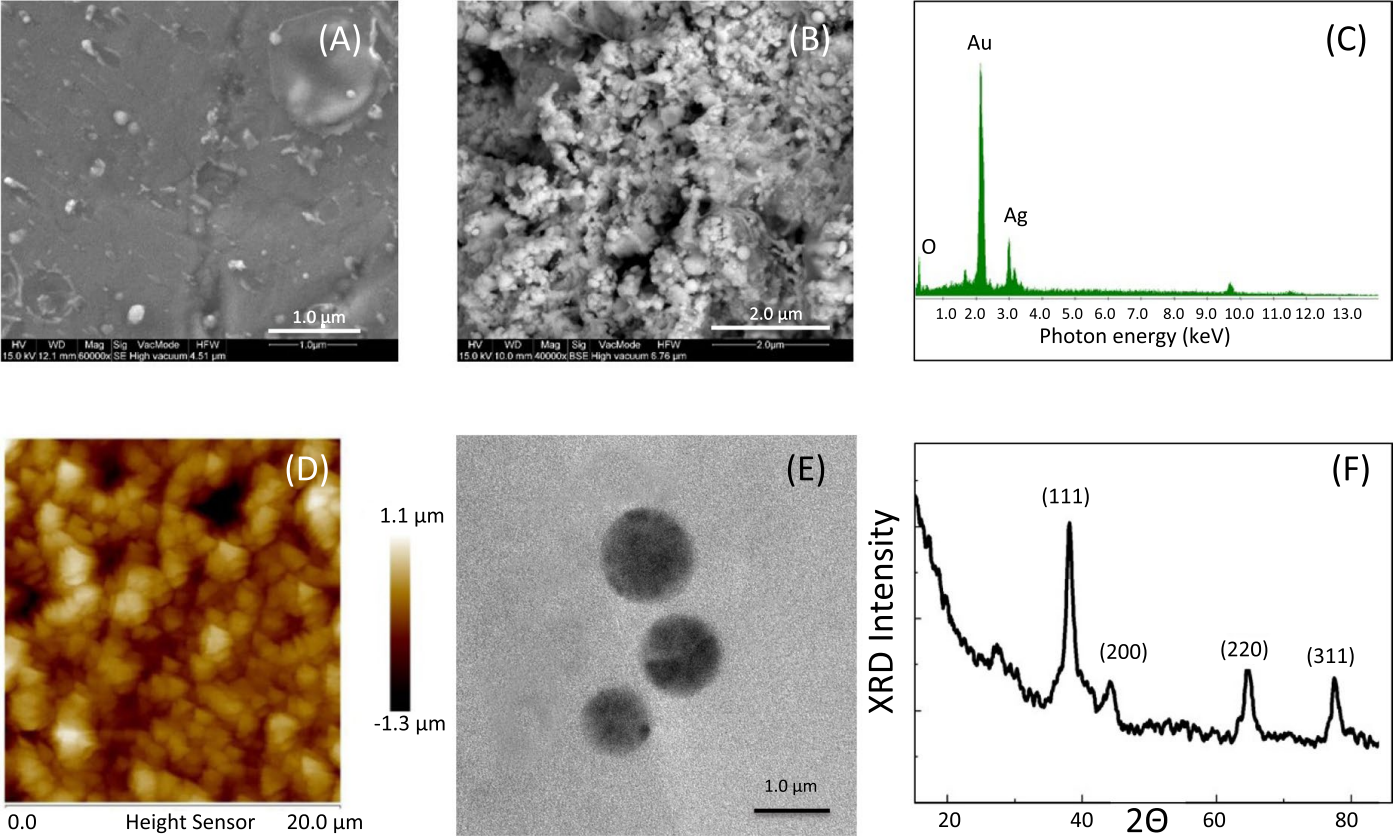
SEM images of the target before (**A**) and after (**B**) irradiation; (**C**) EDX images of the silver target after irradiation; (**D**) AFM and (**E**) TEM image and (**F**) XRD spectrum of gold microcrystals deposited onto the silver substrate.

Our predictions were confirmed by experimental results for the above-mentioned optimum distance condition between the proton source and the irradiated gold sample, i.e. a distance between 2.3 cm to 2.7 cm. Scanning Electron Microscope (SEM) images (see Fig. 3A and B for an image before and after irradiation) indicate a strong erosion of the gold target, which appears to be completely melted after the proton irradiation with many craters having dimensions ranging from hundreds of nm to few μm. The initially smooth surface of the gold target becomes highly porous and rough. The target morphology analysis performed after the irradiation clearly indicates a strong thermal shock with the formation of a fluid state after irradiation, where the atoms are free to move and rearrange in the matrix or can be ejected from the surface. The disordered arrangements of atoms on the surface and the complete absence of micro- or nano-structuration on the gold target indicates a cooling time lasting longer than a few ns, in accordance with the theoretical previsions.

**Figure 4:**
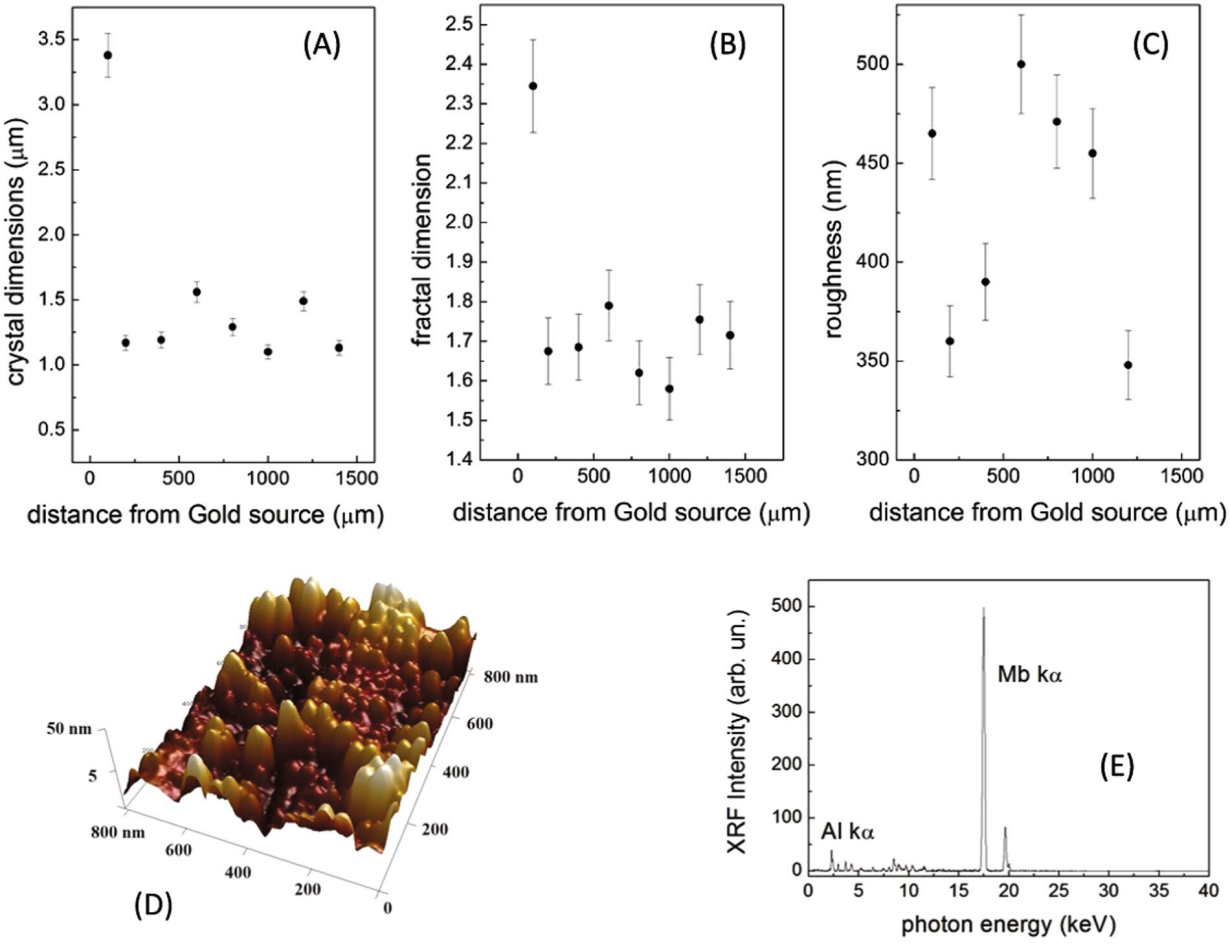
(**A**) Crystal dimensions, (**B**) fractal dimensions, and (**C**) surface roughness of the gold microcrystals deposited onto a silver substrate evaluated from AFM images; (**D**) AFM image and (**E**) XRF spectrum of aluminum microcrystals deposited onto the molybdenum substrate.

Atomic Force Microscope (AFM) images (Fig. 3D) taken from the silver target surface located close to the irradiated gold target show the formation of a microstructure composed by triangular particles with dimensions of about 1 μm (see Fig. 4A). The mean size of the microstructures depends on the distance from the gold source and, for distances above 200 and up to 1500 µm, fluctuates between 1.2 up to 1.5 µm. However, within a more restricted area of 5 × 5 µm (area of the AFM), the precision of the mean size increases, reaching variances around the mean value of about 5% as computed by the AFM software (see methods). A detached gold particle solved in distilled water is shown in the TEM image (see Fig. 3E). The X-ray diffraction (XRD) data in Fig. 3F show the presence of gold octahedral crystals with triangular faces (observed by the AFM tip) with orientation (111), (200), (220), and (311). The crystallite sizes for each mono-crystal visible in the XRD pattern, when using the Scherrer equation with a K factor of 0.9, an X-ray wavelength equivalent to the Cu K_α_, the line broadening and Bragg angle for each band evaluated using a Gaussian fit obtained with the data analysis software (Origin 8.0), are all in the range of a few nm, varying from about 2 nm for the (101) up to 9.24 nm for the (111) line. The micro-crystals visible in the AFM images are consequently an aggregation of the small crystals obtained in the plume.

Energy Dispersive X-ray Analysis (EDX) measurements taken on the micro-structured surface of the silver target (Fig. 3C) show a bulk composition consisting in a thick layer of gold micro-particles deposited on the silver surface. The high gold percentage (60% respect to 30% of silver, the remaining 10% being oxygen) confirms that the triangular structures detected on the silver surface are composed by gold atoms. Analysis of the micro-particle morphology in different locations for different distances between the gold and silver targets shows the formation of a microstructure with constant dimensions (Fig. 4A), fractal dimension (Fig. 4B) and roughness (Fig. 4C) on the silver surface, indicating that a uniform distribution of particles nucleate in the plasma plume, particles that cover on the silver targets a surface of about 4 × 6 mm. From this we can deduce that our LDPA method is able to generate, with a single sub-ns laser shot, micro-structured particles over surfaces measuring several tens of mm^2^.

All the obtained structures show a pure chemical composition without presence of impurities, and a regular distribution of particles in terms of shape and dimensions, similarly to what can be obtained with conventional methods, such as the Laser Ablation Synthesis in Solution (LASiS)^34,35^. Differently from LASiS or PLD (where the time required to synthesize the structures is in the range of tens of minutes and the setup requires more complicated *in-situ* or *ex-situ* control methods^36^), our LDPA method has the advantage to be quicker and more precise. Moreover, LDPA has the possibility of controlling *a-priori* the particle dimensions simply by changing some proton setup parameters (i.e. the distance between proton source and target, the proton beam’s duration or the driving laser’s power). With the classical laser-based techniques (i.e. LASIS or PLD), the control over the synthesized structures can be well-achieved only *a-posteriori*, i.e. after microscope analysis, or *real time*, using UV-plasmonic spectroscopy^34^. A drawback of our method is that the nanocrystals need to be detached from the surface, which, compared to LASiS, requires an additional step before utilizing the nanocrystals. Up to current, the detachment requires some time in order to be fully completed, and requires an optimized choice for both, the materials for depositing the nanocrystals, and the detaching solvent. With respect to the PLD, our method is much faster and the nanocrystals aggregate in the plume, which avoids problems linked to the mobility and aggregation of atoms on the surface (typical of the PLD).

Finally, we repeated the experiments irradiating an aluminum (99% purity) sample located at 2.5 cm from the proton source, and depositing aluminum particles onto a Molybdenum substrate. Hydrodynamical simulations indicate a temperature of about 1750 K for the aluminum target surface, while morphological and chemical analysis, illustrated in Fig. 4D–E, shows the deposition, on the Molybdenum surface, of a nanostructure composed by hemispherical particles with dimensions of the order of 150 nm. This confirms the results obtained with the gold target, indicating the possibility of growing nanostructured surfaces tuning the irradiation conditions.

In conclusion, in this paper we develop a novel Laser-Driven Proton Beam Ablation method for the micro-structuration of surface materials. Experiments using a laser-driven proton beam impinging a gold target show the formation of crystalline gold microstructures on a neighboring silver surface positioned at a variable distance (0–4 mm) from the gold target. The microstructure is composed of octagonal crystals, with uniform dimension (1.2 μm), high precision and density in the entire ~24 mm^2^ area covered by the plasma plume. Experimental findings are confirmed by Monte Carlo simulations, which show that our laser-generated proton beam reproduces ideal conditions for a controlled growth of micro-crystals. In the irradiated gold bulk we are able to reproduce temperature, density, and pressure conditions typical for explosive boiling. The material detachment from the bulk surface, followed by the formation and expansion of a plasma plume, generate the nucleation and aggregation of gold crystals and their deposition onto the surrounding targets.

## Supporting Materials – Experimental Methods

### Laser experiment and diagnostics

**Figure 5:**
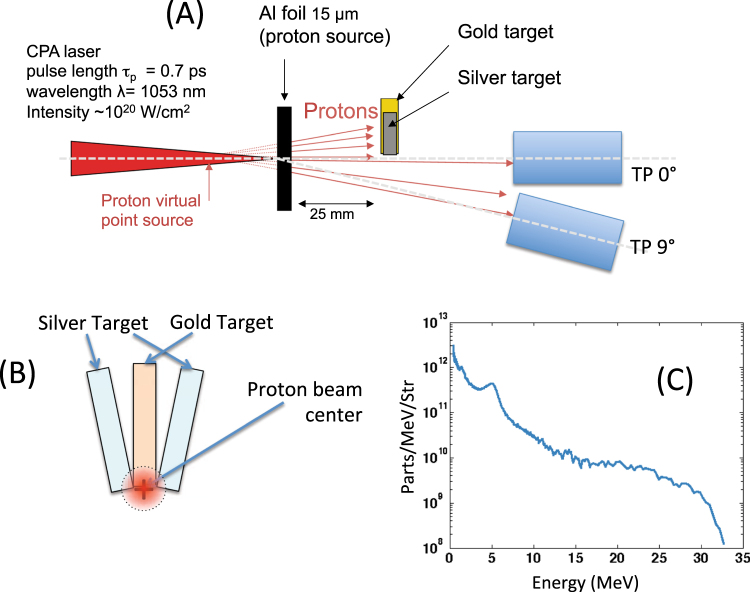
(**A**) Experimental set-up; (**B**) frontal view of the setup; (**C**) typical proton spectrum measured during the shots.

The experiment was performed using the TITAN laser facility located at the Lawrence Livermore National Laboratory (LLNL), Livermore (USA). The experimental set-up is shown in Fig. 5. A laser with energy E~220 J, pulse duration τ = 700 fs, wavelength λ = 1.053 µm, 8-10 µm focal spot diameter (FWHM), and intensity I~10^20^ W/cm^2^ was used for interacting with an aluminum 15 um target in order to accelerate protons in the laser-forward direction using the TNSA^30^ mechanism. The Amplified Spontaneous Emission (ASE) has been measured to be < 10^-6^ in contrast, i.e. delivering ~10 mJ in energy. The protons stemming out from the aluminum foil were impinging into a second target, made of gold (gold 99.9% and 100 µm thickness manufactured by Goodfellow) which was placed on axis at a distance of 2.5 cm (Fig. 5A). Next to the secondary gold target we placed two silver targets with variable distance from the gold target. Both silver targets were positioned radially (see Fig. 5B).

As diagnostics, we used two calibrated Thomson parabolas (TPs) and spectrometers located at 0° (TP 0 °) and 9 ° (TP 9 °) with respect to the main pulse laser axis to measure the forward generated proton spectrum. The TPs were placed respectively at a distance of 690 and 565 mm from the proton source (distance to the entrance slit). Proton spectra measured by the TPs were readout in an absolute manner^37,38^ using Image Plates (BAS-TR 2025 from Fuji Photo Film Co. Ltd) that were analyzed using a FUJIFILM FLA-7000 reader. Additional measurements of the proton spectra were obtained using Radio Chromic Films (RCF) of the type HS that allowed obtaining a beam spatial distribution. During the shots, the target was placed occupying only half of the proton beam so that the TP could readout the spectrum using the other half of the proton beam.

### Interaction simulations

The interaction between the laser-generated proton beam and the aluminum foil was modeled using a two-dimensional Monte Carlo code and using for the proton stopping power a model as indicated in ref.^39^. We have inserted into the code the proton source as obtained in the same experimental conditions and as measured during the shots (an example of proton spectrum is shown in Fig. 5C). The laser-generated proton beam was modeled as the projection of a proton point source with diverging rays at a certain distance, in order to have a proton source of diameter 50 µm. This was modeled similar to what found in ref. ^5^ and considering the laminarity of the beam as indicated in ref.^6^

The divergence half-angle of the proton rays (α) has been adjusted depending on the considered proton energy as obtained in ref.^40^. Within the opening angle, all particles were uniformly distributed. Several simulations were run in order to find the most suitable distance in order to identify the optimum distance between proton source and the second gold target for catalyzing the above-described process.

### Morphological analysis

Morphological analysis on the nanostructured surfaces was conducted by SEM and AFM microscopies. AFM images were obtained using an ICON AFM microscope from Bruker working in tapping mode. Each image was taken with a resolution of 1024 × 1024 pixels and a frequency of about 1 Hz. Shape and dimensions of NPs were analyzed conducting a statistical analysis on about 300 nanoparticles collected in several AFM images. For each sample, we scanned several areas in a window of 500 nm × 500 nm, 1 μm × 1 μm and 5 μm × 5 μm. The images were elaborated using the Nano scope software (1.40 version from Bruker) to obtain a 3D structure and the particle volume using the Bearing analysis. The radius of each particle was evaluated assuming that the volume of a spherical particle is conserved during both, deposition process and interaction, with silicon substrate and/or AFM tip. SEM images were taken under a STEREOSCAN SEM microscope working with an energy of 20 keV.

Crystallinity characteristics of the surfaces were investigated by X-Ray Powder spectroscopy (XRD), using a monochromatic Bruker XRD spectrometer working with the Cu K_α_ line and using a 2Θ configuration at 3 ° of incident X-Ray beam to analyze the first 10 nanometers of the target surface. XRD spectra were analyzed with the EVA software for checking the crystallinity. A Gaussian model fit was used to evaluate the band centers and the full width at half maximum (FWHM) in order to obtain the crystallinity size. The conversion from FWHM to standard deviation was performed using the conversion formula SD = FWHM/2.335.
